# Design of a Flexible Weight Sensor Using Optical Fibre Macrobending

**DOI:** 10.3390/s23020912

**Published:** 2023-01-12

**Authors:** Adam C. Funnell, Peter James Thomas

**Affiliations:** NORCE Norwegian Research Centre AS, 5008 Bergen, Norway

**Keywords:** optical fibre sensor, optical fibre macrobending loss, flexible weight sensor

## Abstract

A flexible weight sensor based on optical fibre macrobending loss, using 1550 nm wavelength light and small fibre bending path lengths is presented. An applied load depresses an impactor layer of cylindrical protrusions into a soft mat covered with optical fibre, causing the optical loss of the fibre to increase. An experimental study of two fibre types, two impactor materials, two impactor designs and a range of protrusion bend radii from 3 mm to 10 mm is shown. For weights greater than 2 kg, a linear response in optical loss (dB) is observed for an applied weight load in kg. The proportionality constant between loss and load, and thus the total amount of optical loss for up to 10 kg of weight load, can be tuned by changing the sensor physical parameters, shown here in ranges from 0.5 dB up to 25 dB.

## 1. Introduction

Optical fibre sensing is an ideal method for measuring parameters such as temperature and strain or even chemical or acoustic signals over long distances [1]. Sensors can be constructed in a fully distributed fashion (using the fibre itself as the sensing medium), or by using point measurements (e.g., gratings etched into fibres) at specific locations along the fibre. Grating based measurement systems such as Fibre Bragg Gratings (FBGs) can translate changes in the fibre’s environment to spectral changes in optical reflectivity, and can even form the direct basis of a pressure sensor [2]. However, FBGs require costly preparation of the fibre by etching in the required gratings, need complex data acquisition using optical spectral properties, and can only respond at particular measurement points along the fibre length. It is desirable to produce sensors which can be constructed from standard low cost optical fibre, and using low complexity electro-optic components with negligible post-processing; a fully distributed system using fibre bending loss to sense applied pressure/weight is therefore appealing.

When optical fibre is bent, the light guided in the fibre core is lost outwards to the cladding and coating, such that the optical transmission is attenuated. Several optical phenomena lead to this result, including macrobending (for fibre bend radii greater than a few millimetres), microbending (for periodic fibre bending structures at sub millimetre scale), and deformation (where localized strains cause the fibre core/cladding cross-section to change shape). Each of these bending effects could form the basis of a mechanical force sensor, where the force of interest acts to bend the fibre.

The macrobending effect arises when light is not fully guided within the core of a fibre, due to the fibre curving around a tight bend radius (mm to few cm). The light is instead coupled into leaking radiation modes which leave the core into the cladding or even the coating and/or external environment [3]. The tighter the bend radius, in general the more light that is lost from the core into the outer layers.

Simple models have been created for this macrobending effect. By extending the model for curvature loss in a waveguide slab into cylindrical co-ordinates, a mathematical expression for the light lost in curved fibre can be found [4]. This derivation relied on the approximation that the field remained the same shape even though the fibre was deformed around a bend; this assumption is invalid for a tight bend radius and thus limits the use of this model to wider bend radii of several mm. Even so, the model has been experimentally validated [5], confirming that most light is lost within the curved region of fibre, not through any coupling effect into leaky modes at the start of a curved area.

Although the model in [4] holds well for fibre comprising only core and cladding layers, it does not explain all of the bending loss variation with the curve radius. In [6] it is shown that the fibre loss not only depends on the macrobending radius, but also shows oscillatory terms which are a function of the fibre geometry and material properties. In particular, the coating layer of a fibre and the wavelength of light have a large influence on the macrobending loss observed. Further assessment is made of the accuracy of [6] in [7], with experimentation showing the scenarios where mathematical simplifications are inappropriate and more complex models are required.

A review of published experimental measurements of the macrobending effect is summarised in Table 1. This review focuses on experimental work which varies fibre and bending properties to assess the important parameters affecting bending loss. The earliest works use shorter wavelengths of transmitted light, while more recent papers use 1550 nm transmission and optical loss measurement, in line with commonly and cheaply available laser sources for telecommunications.

A notable gap in this review is a bend loss configuration, using 1550 nm laser light combined with very small bend lengths (<10 mm), being smaller than the bending radius and turn length. It is this combination that will be exploited in the sensor design presented in this work.

Since the macrobending effect relies on millimeter scale radius changes, it could form the basis of a useful tool for the measurement of weight, pressure or motion. Furthermore, as the quantification of loss requires only the measurement of optical intensity, rather than coherent/phase or other optical effects requiring significant post-processing, macrobend sensors are ideal for low power, low cost applications. Macrobend sensors can also be designed to operate over large sensing lengths (e.g., 0.1 to 10 km), and allow for the sensor to be used in sensitive or hazardous environments where electrical and electronic devices are forbidden.

Although the optical fibre material in all works surveyed in Table 1 is single mode optical fibre made from glass, it is notable that plastic optical fibre can be just as suitable for bending transmission loss applications. Plastic optical fibre is low cost, robust and easy to couple and manipulate. It can display similarly reliable changes in optical loss with variation in the fibre bending radius [16]. A sensor using a macrobending effect on a plastic optical fibre bonded to a deformed beam is described in [17]. Weights hung from a beam caused the bending of a plastic optical fibre, and the resulting optical loss can be used to determine the applied weight. While [17] confirms the option of plastic optical fibre as a material, the implementation targets a very different application than the sensing mat proposed here.

A fibre optic pressure sensor is created in [18], using a multimode fibre passing between two pressure plates which deform the fibre through bending. However, only up to 2 N of force was applied to this sensor before saturation. In [19], an optical fibre bending sensor is used to measure the weight of vehicles driving over a mat containing optical fibres in between grooved rubber plates. While there is a significant variation of the optical intensity passing through the fibre with applied load, there is no attempt made to calibrate the sensors for a quantifiable measure of the sensor weight response.

In [20] a mass measurement scale was built by laying single mode fibre in between a fixed lower set of 8 mm radius cylinders, and a further set of cylinders suspended from a weighing platform mounted on springs. By adding weights to the upper platform, the fibre was bent in between the two rows of cylinders, and the macrobending loss could be read. The system functioned for loads up to 2 kg, but showed poor linearity and repeatability, and saturated for higher applied loads.

Optical weight sensing scales were also constructed in [21], by placing a multimode step index fibre in between a periodic structure of wires to induce bending when load is applied. The sensor was tested for loads up to 100 kg, and careful choice of both the radius of the impacting wires and the periodicity of their impact upon the fibre was necessary to ensure a linear response.

This work presents a simplified optical macrobending sensor design with a flexible construction, where a simple base mat made of a soft foam material has optical fibres directly adhered to the top surface. This design targets future applications where a sensor mat could be placed on rough terrain without requiring expensive ground levelling work or foundations. The optical transmission loss from macrobending relies only upon the bending of the top surface, so the thick foam mat provides cushioning from small stones and debris. If the mat is placed on large scale uneven ground (e.g., over a step, large rock, or on a slope), a calibration in-situ could measure the unloaded fibre transmission loss due to the bumpy terrain alone. Any further loss measured would then be due to applied mass, on any surface type.

This publication specifically focuses on design parameters of the sensor mat which directly affect the macrobending loss. Two types of fibre are considered: standard telecommunications single mode fibre, and fibre designed for a lower single mode cutoff wavelength. The work analyses several different designs for an “impactor” element to bend the fibre, and different types of optical fibre, before drawing conclusions and recommendations for future sensor designs.

## 2. Materials and Methods

**Figure 1:**
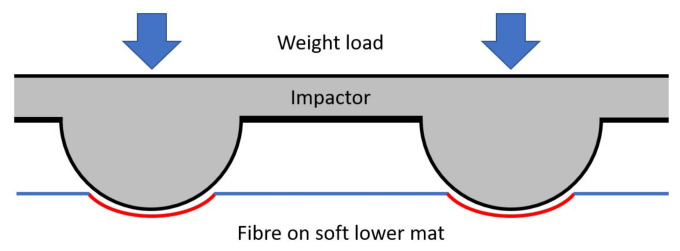
The overall sensor design, showing how weight applied from above pushes the impactor to deform the bottom soft mat and fibre. The bent path length of the fibre is shown in red.

This sensor is designed as a flexible weight-measurement device e.g., it could be used anywhere to measure the weight of objects without needing to be placed on a perfectly flat or horizontal surface. A mat made of soft rubber foam is used as a base, and has optical fibre adhered to its upper surface. A second flexible mat layer, called the “impactor” in this design, is a rubber mat with protrusions pointing downwards towards the optical fibre. When weight is applied on top of the impactor layer, the protrusions impact downwards into the lower mat and fibre. Figure 1 shows the overall sensor design.

When under load, the soft lower mat will deform, and pin the fibre against the impactor, such that the fibre matches the curvature and bend radius of the protrusions. The path length of the bend will then be determined by the vertical displacement of the impactor into the fibre and lower mat below; increased load will give a longer bent path length and thus increase the measurable optical loss. A nitrile butadiene rubber (NBR) foam mat of 1 cm thickness, and a shore A hardness of 36, was used as the soft base layer in this experiment.

**Figure 2:**
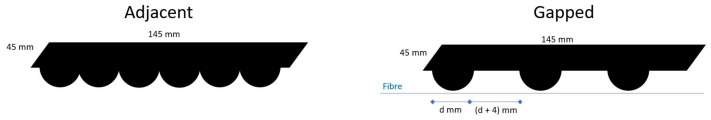
Impactor construction methods, showing the size and spacing of the adjacent and gapped styles of impactor.

All impactor protrusions were made by attaching either cylindrical polyvinyl chloride (PVC) plastic or cylindrical NBR materials to the upper rubber mat, so that the bend radius imposed on the fibre could be well defined around the circular impactor profiles. The impactors were created in two forms: “adjacent” where successive round impactor materials were placed directly next to each other; and “gapped” where a space is left in between successive impactors. Figure 2 shows the two different styles of impactor. In the gapped impactor design, the gap between each impactor is set at the diameter of the cylindrical material plus 4 mm.

It is imperative to securely fix the optical fibres to the surface of the soft bottom mat along their entire length. This ensures that the fibres return to a straight alignment after each deformation from an impactor, and prevents lateral movement or curling during relaxation. Adhesive tape was used in this experiment to secure the fibres to the mat. A future improvement to the design could consider embedding the fibres within the foam mat structure. This could enhance reliability in field applications, and potentially make the sensor easier to manufacture at scale. However, any non-uniformity in the deformation of the mat (between the applied mass and the fibre itself) would also cause non-uniformity in the fibre bending. By using only a thin tape layer in these experiments, the impactor curvature has direct influence on the fibre bending. Varying the impactor curvature radius thus has the defined effect on the fibre in the experiments shown here.

**Figure 3:**
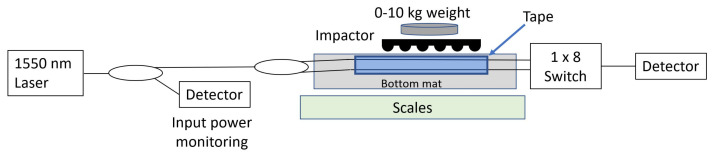
The experimental setup used for the measurements, showing the light passing through the impactor region within both fibre optic cables.

Figure 3 shows the full experimental setup for this investigation. A continuous wave (CW) laser source at 1550 nm was connected to a 3 dB coupler, with one output of this coupler sent to a photodetector to monitor input laser light levels so that source intensity fluctuations could be subtracted from the results. The other output of the 3 dB coupler was then split further, so that the laser source was sent through two different fibre types simultaneously: standard single mode fibre (“SSMF”) for telecommunications, compliant with ITU-T standard G.657; and Thorlabs SM980-5.8-125 fibre designed to have a single-mode cutoff wavelength around 980 nm, well below the 1550 nm used in this experiment. This “low cutoff” fibre is only weakly guiding of the fundamental mode at wavelengths much higher than the cutoff point, and thus greater optical macrobending loss is expected in this fibre than in the telecommunications SSMF.

Both fibres passed under the 14.5 cm long impactor region twice (after an approximately 5 cm radius bend to reverse direction, considered to cause negligible loss compared to the smaller radii under investigation). Since each individual protrusion would only cause a short deformation path length, this double pass ensures that the maximum number of contact points with impactors are imposed, thus increasing the measurable effect.

**Figure 4:**
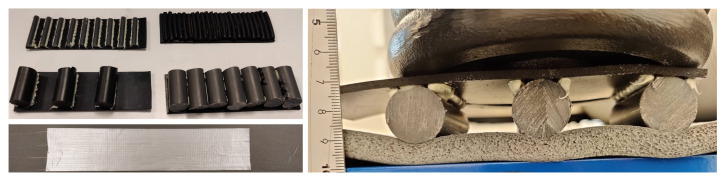
The experimental investigation of the sensor design. Top left, examples of the impactor samples created, showing both soft rubber and hard PVC plastic impactor materials, and gapped and adjacent designs. The white substance around the gapped impactors is supporting adhesive. Bottom left, the two different fibres taped to the foam mat. Top right, an example of the bending of the mat upper surface, and therefore also the taped fibres, under an applied weight load on top of an impactor sample.

Photographs of the experimental sensor design are shown in Figure 4. The photographs show how the impactors were supported by a generous layer of adhesive to ensure no lateral movement of the impactors when under a weight load from above, along with both types of fibre being taped to the mat surface simultaneously, but without interference between them. Figure 4 also shows an example of the bending deformation under an applied weight load; mass applied to the upper impactor surface clearly deforms the top surface of the mat with the optical fibre adhered to it.

The entire sensor was placed on top of a set of weighing scales, so that the weight load placed on top of the impactor could be measured accurately during characterisation.

## 3. Results

**Figure 5:**
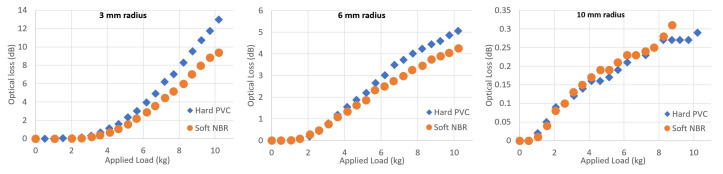
Experimental data comparing hard PVC and soft NBR material forming the macrobend sensor impactors. SSMF was used for all datasets.

Using a set of gapped impactors and considering only the macrobending loss of SSMF, a comparison was made between using hard PVC material or soft NBR material to form the impactor protrusions. Figure 5 shows the difference between the two impactor material types, for three different bend radii.

There is good agreement between the two material types for all of the impactor radii measured. Since the measurement for each material required independent experiments, the agreement between materials for each radius supports the conclusion that the optical loss arises from macrobending effects due to the bend radius imposed on the fibre. For 3 mm radius, the hard PVC material causes up to 4 dB greater loss at 10 kg of applied load, compared to the soft NBR material. It is likely that the soft NBR impactor material deforms under a large weight load, and this would therefore increase the effective bend radius applied to the fibre, in turn reducing optical loss. However, this effect is less noticeable for a large bend radius, since any small deformation would cause a smaller fractional change to the bend radius under high load values.

**Figure 6:**
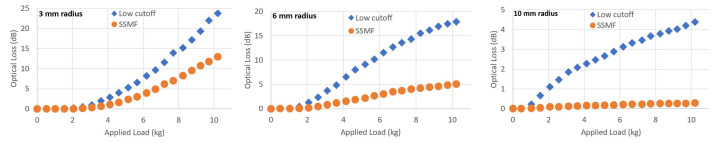
Experimental data comparing bend loss versus load characteristics for standard single mode fibre (SSMF) and low cutoff wavelength fibre. In all cases the fibres were resting on a soft NBR mat, and PVC gapped impactors were used.

A direct comparison can be made between the two types of fibre used in these experiments. Using hard PVC gapped impactors, measurements were made of the optical loss experienced by both fibres (the fibre optic switch shown in Figure 3 was used to measure both fibres in turn during the same impactor sample experiment). Figure 6 shows the loss for each of the fibres, for three different bend radii.

The low cutoff fibre shows consistently higher optical loss than SSMF, for all of the impactor radii studied. The effect is particularly noticeable for 10 mm radius. Since fibre optics are more weakly guiding of light as wavelength increases from the cutoff wavelength, this is an expected effect. If a weight sensor is designed over a large length or area, using a long length of fibre, the expected optical loss from low cutoff fibre must be checked to ensure that it does not exceed the available optical budget offered by the laser intensity and detector noise level. However for small measured loads, the optical loss response for SSMF bent around 10 mm protrusions may be too small to measure with precision.

**Figure 7:**
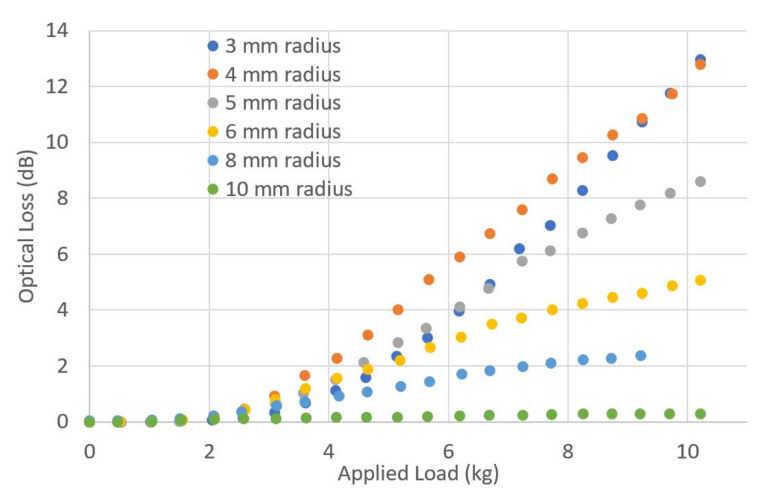
Varying the impactor radius to assess the impact on the optical loss of SSMF under applied load. All impactors were made of hard PVC material.

To assess how the size of the impactor radius affects the optical loss, a series of gapped impactor samples were made using circular hard PVC material, of 3, 4, 5, 6, 8 and 10 mm radius. The optical loss through SSMF, measured as load was applied to the top of the impactor, is shown in Figure 7.

In general, the tighter the bend radius that an optical fibre is subjected to, the greater the optical loss that it will incur [22]. However, Figure 7 shows that the smallest bend radius of 3 mm caused less loss than the next radius of 4 mm. In [6,7] and others, similar effects are observed, where the fibre coating geometry and materials cause such large oscillations in the macrobending loss that the bend radius is not the only factor. The oscillation in optical loss comes from the triple layer core-cladding-coating behaving as a thin-film filter for optical radiation that leaks out of the core around a bend [6]. Precise information on the SSMF properties to permit modelling (e.g., core diameter, coating refractive index) is proprietary information and could not be shared with us to permit direct comparison with a simulation.

**Figure 8:**
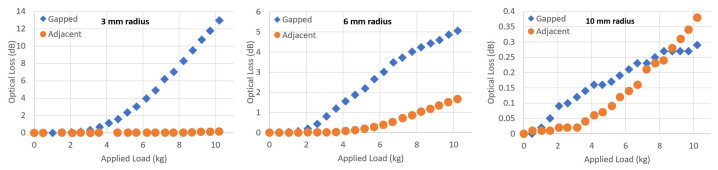
Experimental comparison of the optical loss when using impactors with gapped and adjacent protrusions. SSMF was used in all cases.

The two different types of impactor construction (gapped and adjacent) produce different amounts of optical loss when they are applied to the fibre and mat with a load on top. Figure 8 shows the optical loss caused by both gapped and adjacent impactors of the same radius (for 3, 6 and 10 mm radius samples) when applied to the fibre on a soft mat.

The different loss for gapped or adjacent impactors are due to the differing path length of the bent fibre. The gapped samples would allow the fibre to bend further around the surface of each protrusion, increasing the overall bend path per protrusion. An adjacent construction, particularly for small radii, is likely to only force a longer length of fibre directly downwards into the soft lower mat, by pushing downwards from each protrusion equally. Although the adjacent impactor samples do have a larger total number of protrusions within each 5 cm × 15 cm test area which was used for the experiments, compared to the gapped samples, the effect of wrapping the fibre further around each protrusion dominates optical loss.

**Figure 9:**
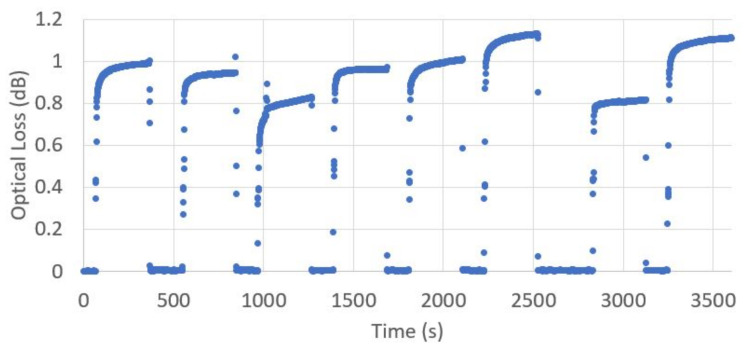
Repeatedly loading and unloading the top surface of an 8 mm radius soft NBR impactor sample, with a 6 kg load. The optical loss of SSMF is shown here as a function of time, where the loading and unloading process was repeated eight times over one hour.

To assess the reliability of this sensor design, and the repeatability of measurements made, an 8 mm radius gapped impactor, made of soft NBR, was repeatedly loaded and unloaded with a 6 kg weight. The optical loss through SSMF was recorded every second, and is shown in Figure 9.

The optical loss at zero load always returns to within ±0.01 dB of the original unloaded value, showing a high degree of reliability and no long term drift in the unloaded state. However, the fluctuation around the optical loss value under load shows a range of ±0.16 dB around the mean value of 0.97 dB. This is a standard deviation of 0.12 dB across the samples measured. This could be partially explained by the nature of the load, which was not uniform over the whole impactor surface area, and a non-zero tolerance in the load centre of mass position for each loading repetition.

The settling time before reaching the final steady state measured value is also of note. Figure 9 shows the time delay for measured optical loss to approach a final steady state value, with 90% of the final steady state value reached in 17.1 s and 99% of the steady state value reached in 134 s on average of the loading events recorded. This settling time places an upper bound on the frequency at which steady state measurements could be made using this system.

## 4. Discussion

For all of the bend radii shown here, there was no measurable optical loss below 2 kg of applied load. This is likely due to the inherent strength of the lower mat, which could support around 2 kg over the area tested without observable deformation. This is expected from the general stress-strain behaviour of polymer foam mats, where only a small linear strain response is expected for small applied stress, up to a critical strain between 5 and 10% above which the strain response becomes large for variation in stress [23]. To avoid this in an operational sensor design, the upper mat could be pre-loaded with the minimum mass required to deform the mat, such that any additional load will always cause a change to the optical loss properties of the fibre. Alternatively, the material properties of the lower mat can be adjusted to ensure that any applied weight load from above will cause a deformation, which in turn will bend the fibre.

However, once the 2 kg load threshold is passed, the optical loss in dB increases approximately linearly with applied weight loading. The proportionality constant between weight and optical loss varies drastically with the physical properties of the sensor e.g., the type of optical fibre, protrusion radius, and protrusion material. For example, a 10 kg load gave 24 dB optical loss for low cutoff fibre and 3 mm radius hard PVC impactor protrusions, while a 10 kg load also only gave 0.3 dB optical loss for any material of gapped impactor in SSMF. For a practical application, the maximum optical loss budget should be determined as the difference between the source intensity and the minimum detector sensitivity. Assuming that the optical loss of this sensor will increase linearly with its length, dividing the loss budget by the desired length will provide the acceptable optical loss per meter. The data shown here, measured for a 30 cm length of impactors, can then be used to determine the fibre type, impactor material and impactor radius to optimise loss over the desired measurement range.

**Figure 10:**
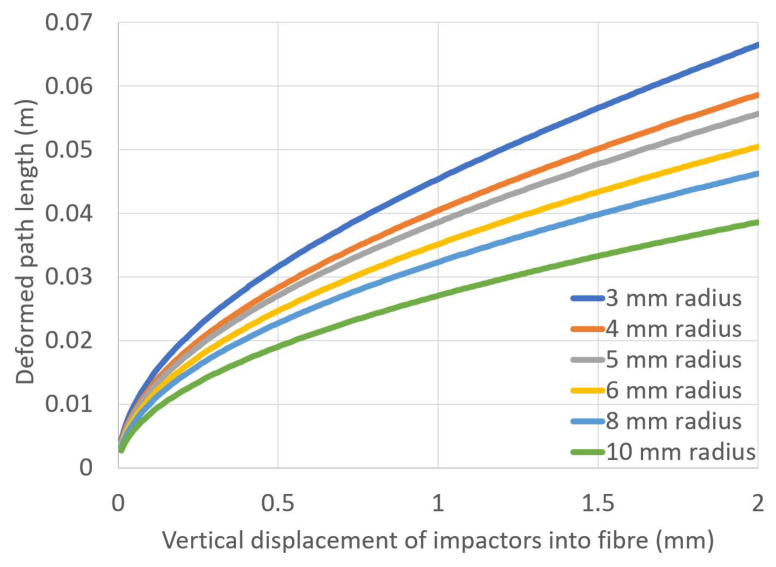
Calculation of the deformed fibre path length as the impactors are brought into contact with the fibre.

The effect of bend radius on macrobending loss must be compared to the increase in deformed path length depending on the number of impactors used on the fibre. The area of the upper impactor surface remains constant throughout, so the number of protrusions downwards into the fibre had to be reduced as the radius of each impactor increased. Figure 10 shows a calculation of the deformed path length for each radius of impactor. This calculation assumes that the fibre bends exactly around the surface of every protrusion as the impactor is displaced downwards into the soft lower mat.

If the applied load on top of the impactors caused the lower mat to deform linearly with the applied mass, the optical loss would be expected to decrease in the same manner with the applied load. However, none of the measured figures in this work display a similar trend, suggesting a more complex relationship. The material properties of polymer foam mats do suggest a linear relationship between stress and strain for small loads (less than 10% strain) [23], but due to the curved protruding shape, the impactor will not be producing an linear increase in stress (pressure) on the mat as the mass load is increased. Further work is required to characterise and/or model the deformation of the soft mat as a function of the applied weight load from above.

The initial results on the choice of hard PVC plastic or soft NBR material for protrusions on the impactors showed very little difference in the macrobending regime (Figure 5). This should simplify the process of manufacturing these sensors at scale; if a soft material can be used to create both a flexible impactor surface, and the protrusions themselves, it should be more easily produced since there is no need to create a composite of both hard and soft materials.

It is promising that over a series of repeated trials, zero applied weight consistently and reliably caused less than 0.01 dB of optical loss. However, the relatively high standard deviation in the measured data points reduces the precision to which weight measurements could be claimed. This could be partially explained by the load not being applied in exactly the same area of the impactor surface every time, which in turn varied how much and how many protrusions bent the fibre. Careful design of the loading surface, such as the position of both the fibres and impactors in 2D space, could result in a mat which has a more even loading profile across the whole surface.

## 5. Conclusions

A design for a flexible weight sensor, based on fibre optic macrobending optical losses was presented. By forming an rubber impactor mat with periodic protrusions, and letting it impact downwards into a soft mat covered with optical fibre, the optical loss of the fibre varies with the weight load applied to the top of the impactor. For applied loads above 2 kg, the optical fibre loss measured in dB increases approximately linearly with the load applied, although the proportionality constant depends entirely on the sensor properties, resulting in optical loss measurements between 0.5 and 25 dB for a 10 kg load.

An experimental study of the parameters to optimise the sensor design was performed. Analysis showed that the fibre (low cutoff or standard telecommunications fibre) has the largest impact on the overall optical loss, followed by the material of the bending protrusions. The impacting protrusions should be left with a gap in between, to allow the fibre to bend freely around each protrusion and increase the bending loss. Careful choice of the fibre and impactor protrusion radius must be made based on the optical loss budget available, and the length over which the sensor will be operational.

## Figures and Tables

**Table 1:** Single mode fibre bending loss experimental measurements.

Paper	Fibre Cutoff Wavelength (nm)	Bend Radii Tested mm	Laser Source Wavelength nm	Fibre Mode Field Diameter (μm)	Fibre Bend Length
Faustini [7]	620 and 850	13.5–27.5	1480 and 1550	Unknown	Half turn
Gauthier [8]	450 and 750	1.2–6.4	632.8	3.1 and 4.0	6.25 um to 30 mm
Harris [5]	Unknown (high NA)	2–16	633, 790 and 1152	Unknown	Quarter turn to 2.5 turns
Kamikawa [9]	1040 to 1420	1.25–8	1550	6.26–10.28	Half turn
Morgan [10]	1232	2.5–7	1220–1660 broadband	9.78	60 to 180 degrees
Murakami [11]	620 and 700	15–55	1150	Unknown	Full turn
Martins [12]	Unknown	1–10	1550	8.2	Unknown
Tangonan [13]	Unknown	1–8	1550	9.1, 8.4, 7.5, 7.3	60 to 180 degrees
Wang [14]	Unknown SMF28	8.5–12	1500–1600	Unknown	1–2 m
Wang [15]	Unknown SMF28	5.5, 6.0 and 6.5	1500–1600	Unknown	One turn

## Data Availability

The data presented in this study are available.
